# Assessing the public discourse on Twitter: Reactions to the JUUL e-cigarettes ban in the United States

**DOI:** 10.18332/tid/184053

**Published:** 2024-02-26

**Authors:** Artur Galimov, Larisa Albers, Tahsin Rahman, Julia Vassey, Matthew G. Kirkpatrick, Jennifer B. Unger

**Affiliations:** 1 Tobacco Center of Regulatory Science, Institute for Health Promotion and Disease Prevention Research, Department of Population and Public Health Sciences, Keck School of Medicine, University of Southern California, Los Angeles, United States

**Keywords:** JUUL, twitter, public opinion, public policy, social media

## Abstract

**INTRODUCTION:**

JUUL is a high-nicotine pod-based vaping device that is popular among adolescents and young adults. On 23 June 2022, the US Food and Drug Administration (FDA) denied authorization to market JUUL, and ordered JUUL Labs to remove products from the US market. The next day, a US federal appeals court temporarily suspended the ban. The mixed public discourse surrounding the FDA ban warrants further investigation.

**METHODS:**

This study examined Twitter data to describe public reaction to these announcements. Posts containing terms ‘JUUL’ and/or ‘#JUUL’ (N=97548 unique tweets) were collected from 23 June to 3 July 2022, from Twitter’s Streaming Application Programming Interface (API). After removing retweets, we used an inductive approach to become familiar with the data, generated a codebook, and conducted a content analysis on a random sample of n=4000 tweets.

**RESULTS:**

A total of 2755 (68.9%) tweets discussed JUUL in the context of the FDA ban. News (n=1425/2755; 51.7%) about the JUUL ban, government distrust (n=588; 21.3%), and individual rights (n=253; 9.2%) were the most prevalent themes. Less commonly discussed themes included inconsistencies between policies (n=174; 6.3%), mentions of switching to other products (n=162; 5.9%), smoking cessation (n=99; 3.6%), and craving for JUUL (n=94; 3.4%). Sentiment analysis of JUUL ban-related posts (n=2755) demonstrated that 1989 (72.2%) tweets were categorized as neutral, while anti-ban posts (n=566; 20.5%) were more prevalent than pro-ban posts (n=200; 7.3%).

**CONCLUSIONS:**

Besides straightforward announcements of the JUUL ban and its suspension, Twitter posts discussed government distrust, individual rights, and policy inconsistencies. While most posts conveyed neutral sentiments, anti-ban posts were almost three times more prevalent than pro-ban posts. Our findings suggest that text-based social media platforms like Twitter may be an effective instrument to understand opinions, attitudes, and beliefs regarding the FDA’s JUUL ban.

## INTRODUCTION

JUUL is a high-nicotine electronic cigarette (e-cigarette) product, known for its sleek design, appealing flavors, and social media-based marketing^1-3^. Since its introduction to the US market in 2015, JUUL has gained popularity among adolescents and young adults and became the most commonly used vaping product in 2019^1,2,4^. Findings from a 2018 national, probability-based sample of participants aged 15–34 years demonstrated that 6.0% self-reported ever JUUL use, while 3.3% reported past 30-day use^5^. The prevalence of JUUL use was significantly higher in younger participants: 11.2% of young adults (18–21 years) reported ever use and 7.7% reported past 30-day use, while 9.5% of adolescents (15–17 years) reported ever use and 6.1% reported past 30-day use^5^.

One possible explanation for the heightened prevalence of JUUL use among adolescents and young adults is the company’s aggressive marketing and social media campaigns tailored toward younger audiences, including social media influencers, branded merchandise, and targeted advertisements^4,6^. Tobacco companies, such as Philip Morris USA (part of Altria, which partially owns JUUL Labs), have a long history of lobbying against regulatory action and funding research to advocate industry positions^7^. For instance, JUUL and other vaping products are often marketed as a ‘safer alternative’ to traditional cigarettes^7,8^. Nonetheless, burgeoning evidence suggests that e-cigarette products are not completely harmless; their potential effects include neuroinflammation, exacerbations of asthma, elevated blood pressure, cognitive impairment, nicotine dependence, and initiation of combustible cigarette use^9,10^. Public health experts have called for greater regulation of e-cigarette products and increased public awareness of their potential health risks^3,4^.

One such regulatory effort occurred on 23 June 2022, when the US Food and Drug Administration (FDA) ordered JUUL Labs Inc. to stop selling and distributing their products on the US market^11^. The FDA denied authorization to market JUUL products, stating that JUUL’s premarket tobacco product applications had not provided sufficient evidence to demonstrate that their products were not toxicologically harmful^11^. Nevertheless, on 24 June 2022, a US federal appeals court temporarily halted the government’s ban, after JUUL filed an emergency motion and appealed against the FDA’s order^12^. Public reactions to FDA’s JUUL ban featured in the news varied, with praise from public health groups and criticism from JUUL users who argued that JUUL was a viable alternative to the purportedly more harmful traditional combustible cigarettes^13^. The mixed public discourse surrounding the FDA ban warrants further investigation, as such analyses can guide tobacco control measures, inform health communication efforts, and help contextualize tobacco use trends among adolescent and young adult populations^14,15^.

Social media is an important tool for examining industry marketing strategies and tracking conversations about emerging topics and products. For instance, prior studies have shown that Twitter data can yield valuable insights into the public discourse on tobacco control measures^14,16,17^. Twitter’s user population includes a variety of demographics, locations, and interests, with 22% of US adults and 32% of teenagers aged 13–17 years engaging with the platform, and 42% of its users logging in daily^18,19^. While social media data (including Twitter) have been previously utilized by researchers to understand organic discussions about JUUL and other tobacco products, its application in analyzing public reactions to the FDA’s JUUL ban remains limited^17,20-24^. The diversity of Twitter’s user base has the potential to provide a broad perspective on the range of attitudes, beliefs, and opinions regarding the JUUL ban. To document the public reaction to the FDA’s JUUL ban announcement, this study examined JUUL-related Twitter posts on the day the ban was announced (23 June), the day it was suspended (24 June) and the next 10 days following the announcement. Identification of major themes in these posts may guide future health communication and regulatory strategies.

## METHODS

Posts containing terms ‘JUUL’ and/or ‘#JUUL’ were collected from 23 June to 3 July 2022, from Twitter’s Streaming Application Programming Interface (API). A total of n=97548 unique posts containing these terms were identified (excluding re-tweets). Similar to previous studies, we randomly sampled a subset of n=4000 posts for the content analysis^20,24^. The authors worked together to become familiar with the data, created a codebook, and identified nine themes using an inductive approach^25^. The unit of analysis was the text of the Tweet, and the aim of this approach was to condense the raw text-based data into a summary format and report the underlying themes evident in the data^25^. This strategy was chosen to ensure that the identification of themes was guided by the data themselves, ensuring they are deeply rooted in the actual content rather than being influenced by preconceived categories.

We identified (Table 1) the following themes: 1) News (divided into the following two subcategories, a. JUUL ban announcement, and b. JUUL ban temporary suspension); 2) Government distrust; 3) Individual rights; 4) Inconsistencies between policies; 5) Switch to other products; 6) Smoking cessation; 7) craving for JUUL; 8) Black market; and 9) Other. A tweet could be classified into more than one theme. In addition, we coded the sentiment of 2755 posts related to the JUUL ban and classified them into two categories: pro-ban and anti-ban^23,24^. Neutral tweets were those that expressed neither pro-ban nor anti-ban sentiments. To establish inter-rater reliability, two authors double-coded a subsample of posts (n=500), with percent agreement ranging 90.2–100%, and Cohen’s kappa ranging 0.73–1.00. The first author served as an arbitrator to resolve discrepancies between the coders.

**Table 1:** Definition for each theme, descriptive statistics, and selected paraphrased twitter posts (N=4000)

*Topic*	*n (%)*	*Definition*	*Paraphrased post*
**Posts**			
Pro-ban	200 (5.0)	Posts that express a favorable or positive opinion or attitude towards the JUUL ban	*‘We applaud FDA’s decision to remove JUUL products from the US market.’*
Anti-ban	566 (14.2)	Posts that express a negative opinion or attitude towards the JUUL ban	*‘I am very upset about JUUL ban! What is next?’*
**Themes**			
1. News	1425 (35.6)	News articles/announcements about JUUL	*‘US FDA officially bans JUUL in the US.’*
a. JUUL ban announcement	839 (21.0)	A news story, podcast, or announcement about the FDA JUUL ban	*‘FDA bans JUUL tied to youth vaping surge US https://…’*
b. JUUL ban temporary suspension	586 (14.7)	A news story, podcast, or announcement about the temporary suspension of the FDA JUUL ban	*‘BREAKING: Federal court blocks FDA’s ban on JUUL sales in US https://…’*
2. Government distrust	588 (14.7)	Mentions of disagreement with or distrust in the government and/or health organizations, including accusations related to Big Tobacco lobbying interests Posts may also reference perceived hypocrisy concerning the JUUL ban	*‘But they want to make JUUL illegal. What a sick government.’* *‘JUUL was banned yesterday. Wonder how much money Big Tobacco lobbied this time!’*
3. Individual rights	253 (6.3)	Mentions of personal freedoms and liberties, including mentions of the right to use JUUL products or the right to make one’s own choices	*‘I lost my right for buying JUUL and having an abortion all in one week!’*
4. Inconsistencies between policies	174 (4.4)	Mentions of accusations regarding the ban on JUUL while other combustible tobacco products (e.g. cigarettes), substances (e.g. marijuana, fentanyl), and guns remain legal	*‘They really ban JUUL before cigarettes.’* *‘The government bans JUUL while giving fentanyl crack to drug users…OMG!’*
5. Switch to other products	162 (4.1)	Mentions of JUUL users potentially switching to other vaping products, tobacco products, or other drugs in response to a ban	*‘US FDA bans JUUL but young vapers are already switching to other novel vaping products.’*
6. Smoking cessation	99 (2.5)	Mentions of the use of JUUL to quit cigarettes	*‘JUUL helped me to quit smoking. Do they want people to switch back to cigarettes?’*
7. Craving for JUUL	94 (2.4)	Mentions of cravings for or addiction to JUUL products, possibly including references to stocking up on JUUL products or searching for them	*‘I am very addicted to these JUUL pods.’*
8. Black market	46 (1.2)	Mentions that the JUUL ban will lead to JUUL sales through illegal channels (i.e. ‘black market’)	*‘The JUUL black market will be crazy.’*
9. Other	1245 (31.1)	Posts in non-English, or any posts that discuss JUUL in a context unrelated to the FDA’s ban on JUUL	*‘I am out of JUUL pods.’*

### Data analysis

Descriptive analyses were conducted to show the prevalence of each theme. To visualize the change in the percentage of themes over time, data were plotted across the study period (from 23 June to 3 July 2022). All Twitter posts in this dataset were publicly available and anonymized, and all analyses adhered to the terms and conditions, terms of use, and privacy policies of Twitter. To further protect privacy, posts were paraphrased; no tweets were reported verbatim. All graphs were created using Stata software (v 17.0; StataCorp). The protocol was approved by the Institutional Review Board of the University of Southern California.

## RESULTS

**Figure 1:**
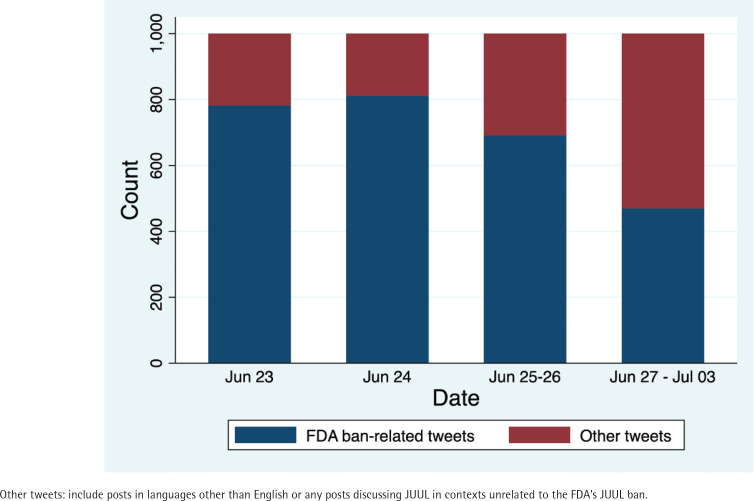
Total tweet counts collected via Twitter’s Streaming Application Programming Interface (API) from 23 June to 3 July 2022, reported by date (N=4000)

Among the 4000 tweets in our sample, 2755 (68.9%) discussed JUUL in the context of the FDA ban. The proportion of these tweets peaked on the day a US federal appeals court temporarily blocked the FDA’s order (24 June 2022) and gradually decreased over the following two weeks (Figure 1). The most prevalent themes among JUUL ban-related posts were *news* about JUUL (1425/2755; 51.7%), *government distrust* (588/2755; 21.3%), and *individual rights* (253/2755; 9.2%). *News* tweets discussed the *JUUL ban announcement* (839/1425; 58.9%) or *JUUL ban temporary suspension* (586/1425; 41.1%). Other JUUL ban-related discussions had relatively lower prevalence in the sample: *inconsistencies between policies* (174/2755; 6.3%), *switch to other products* (162/2755; 5.9%), *smoking cessation* (99/2755; 3.6%), craving for *JUUL* (94/2755; 3.4%), and *black market* (46/2755; 1.7%). Sentiment analysis of JUUL ban-related posts (n=2755) demonstrated that most posts were neutral (1989; 72.2%), while *anti-ban* posts (566; 20.5%) were more prevalent than *pro-ban* posts (200; 7.3%). Descriptive characteristics of the 4000 tweets and examples of paraphrased posts are presented in Table 1.

**Figure 2:**
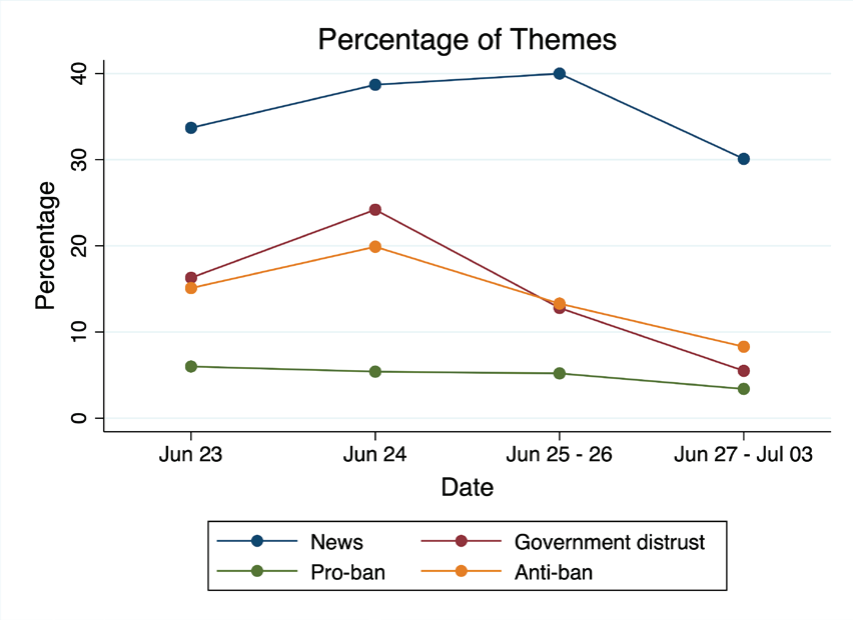
Percentage of tweets by date featuring the most common themes (news, government distrust) and sentiments (pro-ban and anti-ban) in FDA JUUL ban-related Twitter posts (N=2755)

Figure 2 shows the percentage of tweets containing the most prevalent (news, government distrust) sentiment themes (pro-ban and anti-ban) as a function of date. The prevalence of tweets with the *news* theme gradually increased and peaked on the weekend after the JUUL ban was announced and then decreased over the next week. The proportion of *government distrust* and *anti-ban* tweets was greatest on the day when a US federal appeals court temporarily blocked the FDA’s order (24 June 2022) and decreased over the next 2 weeks. The prevalence of *pro-ban* posts remained relatively stable over time.

## DISCUSSION

This study documented public reactions to the JUUL ban announcement and suspension on Twitter by collecting and summarizing 4000 posts that contained the word ‘JUUL’ or the hashtag ‘#JUUL’. In addition to the *news* theme featuring the FDA’s JUUL ban announcement and its temporary suspension, other common topics included *government distrust*, *individual rights*, *policy inconsistencies*, and mentions of *switching to other products*. While most posts conveyed neutral sentiments, *anti-ban* posts were almost three times more prevalent than *pro-ban* posts.

Our findings demonstrated that among all JUUL-related posts in our sample, almost 70% discussed JUUL in the context of the FDA ban. Furthermore, the level of discussion was the most prevalent when the ban was announced and on the day a US federal appeals court temporarily blocked the FDA’s order (i.e. 23–24 June 2022), then it gradually decreased over the subsequent two weeks. Collectively, these results suggest that social media data (and Twitter specifically), can be an effective tool in monitoring the public’s reaction to tobacco-related policies^16,17^. Future public health campaigns could utilize Twitter data to shape more effective tobacco control strategies. For instance, using the hashtag #JUUL may help identify individuals opposed to the ban, to whom current scientific findings on adverse effects of JUUL use and its association with increased combustible smoking initiation among youth could be disseminated.

*Government distrust*, *inconsistencies between policies,* and *individual rights* were prevalent themes in our study. These findings align with earlier Twitter studies that evaluated the reactions to a proposed menthol ban on cigarettes and a potential ban on flavored e-cigarettes^16,17^. Many users expressed skepticism towards the JUUL ban and suggested that it was motivated by the financial interests of tobacco companies. Furthermore, *anti-ban* posts were almost three times more prevalent than *pro-ban* posts. This is concerning because it indicates a lack of trust in public health efforts to regulate harmful tobacco products^26^. Given that tobacco companies, including Altria, have a long history of employing policy front groups to support their position and lobbying through social media campaigns^6,7^, our findings might also be indicative of a concerted campaign orchestrated by the tobacco industry. Further research evaluating the causes and consequences of distrust in public health policies is needed to fully understand this phenomenon and develop strategies for addressing it. Similar to previous Twitter studies, some users argued that the government’s focus on regulating vaping products is misplaced, and should be directed towards more harmful substances like heroin, methamphetamine, or cocaine^16^. Nonetheless, a growing body of research suggests that e-cigarettes are not harmless^9,10^. Hence, despite the challenges and criticisms, the JUUL ban remains an important public health policy.

Using JUUL for *smoking cessation* was another important theme. JUUL has promoted its products as a safer alternative to combustible cigarettes, so it was unsurprising that our study captured cessation-related discussions^8^. While some users may report successful cessation with JUUL, these products lack the evidence base for smoking cessation and are not FDA-approved for this purpose^11^. *Craving for JUUL*, *mentions of switching to other products,* and *black market* were also notable themes in this study. This implies that individuals who use JUUL products are highly nicotine dependent and have difficulty quitting nicotine^21,27^. It is critical to ensure that implementing tobacco-related policies will not lead to unintended consequences (i.e. switching to potentially more harmful products, black market). Overall, the findings highlight the importance of reaching out to nicotine dependent individuals and providing them with information on evidence-based tobacco cessation programs. Twitter, as a widely used social media platform, provides a unique opportunity to reach out to JUUL users and engage them in discussions around tobacco cessation^28^. By leveraging Twitter to reach out to JUUL users who are experiencing cravings or addiction, we can provide them with the support and resources they need to quit tobacco and improve their overall health and well-being.

### Limitations

This study only considered posts from publicly accessible accounts, and therefore may not reflect the attitudes of Twitter users with private accounts. Findings may not generalize to other social media platforms or other time periods. Our findings may not reflect the opinions of all Twitter users or the broader US population, particularly those who do not use Twitter. Data included English-language tweets, which precludes our understanding of reactions from non-English-speakers. This study focused on the text of the posts but did not analyze any website links or images that were attached to them. It is possible that more themes would have emerged had we analyzed this additional content.

## CONCLUSIONS

Discussions on Twitter related to the JUUL ban primarily feature news-related themes (i.e. ban announcements and suspensions); however, topics of government distrust, individual rights, and policy inconsistencies are also common. Most of these tweets convey a neutral sentiment, although anti-ban posts are more prevalent than pro-ban posts. Overall, our findings suggest that text-based social media platforms like Twitter may be effective tools for assessing attitudes, beliefs, and opinions regarding the FDA’s JUUL ban. Future studies examining the characteristics of the users who posted about the JUUL ban, and how these characteristics relate to the content and sentiment of the posts, are warranted. Additionally, public health campaigns could utilize Twitter data to shape more effective tobacco control and health communication strategies.

## Data Availability

Data will be made available upon reasonable request to the corresponding author.
